# Transferrin-conjugated liposomes loaded with novel dihydroquinoline derivatives as potential anticancer agents

**DOI:** 10.1371/journal.pone.0186821

**Published:** 2017-10-31

**Authors:** Mengqiao Wang, Robert J. Lee, Ye Bi, Lianlian Li, Guodong Yan, Jiahui Lu, Qingfan Meng, Lesheng Teng, Jing Xie

**Affiliations:** 1 Jilin University, College of Life Science, Changchun, Jilin, China; 2 Division of Pharmaceutics, College of Pharmacy, The Ohio State University, Columbus, Ohio, United States of America; University of South Alabama Mitchell Cancer Institute, UNITED STATES

## Abstract

A series of 1,2-dihydroquinoline derivatives were synthesized and evaluated for cytotoxicity in HeLa, Hep G2 and 6HEK-293T cell lines. EEDQ2 was identified as a promising anti-cancer agent with low IC_50_ in HeLa (18.55μg/ml) and Hep G2 (14.53μg/ml) cells. For improving the antitumor activity and tumor selectivity of EEDQ2, we prepared transferrin (Tf)-modified liposomes (LPs) to deliver EEDQ2. When HeLa and Hep G2 cells were treated with LP-delivered EEDQ2, the ROS level was significantly enhanced, and mitochondrial membrane potential was reversed. Tf-LPs improved cell uptake of EEDQ2 by about 3.7 times compared with non-targeted LPs. These data suggest that Tf-LPs delivering EEDQ2 is a promising strategy to treat cancer.

## Introduction

Cancer is a complicated genetic disease that progresses via cell signaling, uncontrolled proliferation, metastasis, and apoptosis[1, 2]. Traditional anti-cancer agents suffer from undesired side effects, narrow therapeutic window and drug resistance. There is a significant demand for effective and selective anti-cancer agents. 1,2-Dihydroquinoline (EEDQ) has a heterocyclic ring with nitrogen atom that can be easily modified to obtain a large number of derivatives with therapeutic activities such as antimicrobial[3, 4], antiviral[5], analgesic[6], antitubercular[7] and anticancer activities[8]. EEDQ derivatives with anti-cancer activity are typically metal complexes[5, 9]. These agents have shown promising antitumor effect. However, this is accompanied by serious side effects. We have synthesized a series of novel EEDQ derivatives as potential anticancer agents[10]. However, low water solubility of novel EEDQ derivatives is a major obstacle to their application. Liposomes are a frequently-used delivery system to improve stability, solubility, therapeutic value and reducing side effects of agents[11]. However, when LPs were used to enhance solubility of EEDQ derivatives, the cell penetration capacity of drugs was adversely affected. The receptor for transferrin (TfR) is ubiquitous expressed in all cells. However, expression levels of TfR in most normal cell is 100-fold lower than that in tumor cells[12]. Here, we used Tf-modified LPs to deliver novel EEDQ derivatives, and showed that this can improve tumor cell uptake and antitumor effect.

## Materials and methods

### Materials

Hydrogenated soy phosphatidylcholine (HSPC), cholesterol (Chol), and DSPE-PEG200 were obtained from Lipoid (Ludwigshafen, Germany). DCFH-DA, a fluorescent dye, was purchased from Nanjing Jiancheng Bioengineering Institute (Nanjing, China). 5, 50, 6, 60-Tetrachloro-1, 10, 3, 30-tetraethyl-imidacarbocyanine iodide (JC-1) sulforhodamine B, holo-human Tf and 1, 2-dioleoyl-sn-glycero-3-phosphoethanolamine, and 7-nitrobenzofurazan-labeled (NBD-DOPE) were purchased from Sigma-Aldrich (St. Louis, MO). 4', 6-Diamidino-2-phenylindole (DAPI) was purchased from Invitrogen Molecular Probes (Eugene, OR, USA). HeLa, HepG2 cell lines were purchased from American Type Culture Collection (ATCC).

### Chemistry

**Fig 1:**
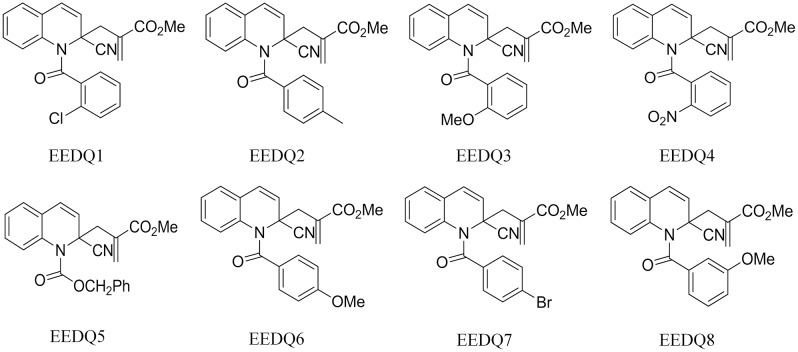
Structures of EEDQ derivatives.

A series of novel EEDQ derivatives were prepared as described previously[13], EEDQ1, EEDQ2, EEDQ3, EEDQ4, EEDQ5, EEDQ6, EEDQ7, and EEDQ8 (Fig 1).

### In vitro anticancer activity

Cytotoxicity of EEDQ 1–8, along with paclitaxel, were studied using human cervical cancer HeLa, human liver carcinoma Hep G2, and normal cells (6HEK-293T) by MTT assay. The compounds of EEDQ 1–8 were dissolved in ethanol and diluted with basic Dulbecco’s modified Eagle’s medium (DMEM). The cells were planted in 96-well plates at 10^4^ cells per well in 100μL DMEM containing 10% fetal bovine serum (FBS), and were incubated at 37°C in a humidified atmosphere containing 5% CO_2_ for 24 h before addition EEDQ 1–8. After addition EEDQ 1–8 to culture another 24 h, 10μL of MTT (5 mg/ml) was added into each well to incubate 4h. The medium was replaced by 150μL DMSO to dissolve formazan crystals. Then, absorbance was measured at 490 nm by a microplate reader. The paclitaxel and basic medium were as the control groups. The results of these tests were analyzed to yield IC_50_ values.

### Preparation of LPs

To enhance the solubility and tumor cell selectivity of EEDQ2, we used an ethanol injection-sonication method to prepare LPs[14]. Briefly, HSPC, Chol and PEG-DSPE at a mass ratio of 36:10:1 were dissolved with ethanol at 10mg/ml. EEDQ2 was added into lipid phase to obtain a final concentration of 1 mg/ml. The lipid phase was fast-injected into PBS (pH 7.4) at 65°C at 1:10 (v/v) under vortex mixing, the solution was sonicated with a bath-type sonicator for 30s to obtain LPs (LP-EEDQ2). The LP-EEDQ2 was dialyzed to remove free EEDQ2 and ethanol. The LPs were then incubated with Tf-PEG-DSPE at a molar ratio of lipid:Tf of 500:1 to prepare Tf-modified LPs (Tf-LP-EEDQ2)[15]. Tf-PEG-DSPE was synthesized by a previously described method [15]. Briefly, Holo-Tf was modified with 5× Traut's to obtain holo-Tf-SH. Then, holo-Tf-SH was incubated with DSPE–PEG–Mal at a molar ratio of 1:10 to yield Tf-PEG-DSPE.

To prepare fluorescence-labeled Tf-LP-EEDQ2, NBD-DOPE (1 mole% of total lipids) was used to label the lipid membrane[16]. In addition, 10 mM sulforhodamine B was also used to label the aqueous phase to prepare double-labeled LPs.

### Characterization of LPs

The size distribution and zeta potential of Tf-LP-EEDQ2 were measured using a Nicomp Zeta Potential/Particle Sizer (Model 380 ZLS, Santa Barbara, CA, USA) by dynamic light scattering in intensity-weighted Gaussian size distribution mode at 23°C with a viscosity setting of 1.333 cP.

The surface morphology of Tf-LP-EEDQ2 was analyzed using a field emission scanning electron microscope (FE-SEM) (JSM-6700F, JEOL, Japan) at 3kV accelerating voltage. The Tf-LP-EEDQ2 suspension was added onto the smooth surface of a silicon wafer and then let dry naturally. The images of FE-SEM were acquired in secondary electron mode[17].

Entrapment efficiency (EE%) is a key parameter of Tf-LP-EEDQ2 that affects the pharmacodynamics and pharmacokinetics of LPs. EE% was measured by ultrafiltration-centrifugation method. Firstly, Tf-LP-EEDQ2 was placed in the cannula of an ultrafiltration centrifuge tube (MWCO 10,000) and then centrifuged at 10,000rpm for 30min at 4°C to separate free EEDQ2 (F_drug_) from encapsulated drug. Secondly, we added 3% Triton X-100 into LPs suspension to release the drug to enable measurement of total EEDQ2 (T_drug_)[18]. Finally, 20μL processed solution was injected into an HPLC system to measure the F_drug_ and T_drug_ of Tf-LP-EEDQ2. EEDQ2 concentration was assayed at 240nm using a Agilent SB-C_18_ column (4.6 mm × 250 mm, 5 μm) at 35°C, samples were separated using a mobile phase containing of methanol/water (60:40) at the flow rate of 1 ml/min.

$$\text{EE}\%=\left({\text{T}}_{\text{drug}}-{\text{F}}_{\text{drug}}\right)/{\text{T}}_{\text{drug}}*100\%.$$

### Drug release profiles

Tf-LP-EEDQ2 was placed into a dialysis bag (MW cut off 8,000–12,000 Dalton) with phosphate buffer solution (pH6.8) containing 0.5% Tween 80 as a release medium[19, 20]. The experimental system was persistently stirred (300rpm) in darkness with 37°C. At fixed time points, 1ml external dialysis medium was collected and replaced with the same amount of fresh dialysis medium. The cumulative percentage of drug release was used to evaluate release profilies.

### Cytotoxicity assay

Measurement of time dependent cytotoxicity of EEDQ2 was carried out using the MTT assay method. The cells were planted in 96-well plates at 10^4^ cells per well with 100μL DMEM containing 10% FBS to culture 24h. After respectively adding EEDQ2, LP-EEDQ2 and Tf-LP-EEDQ2 into wells, the cells were cultured additional 2, 6, 12, 24, or 48h at 37°C in humidified atmosphere containing 5% CO_2_ at 37°C.

The dose dependent cytotoxicity of EEDQ2 preparations were also measured using the MTT assay method. Cells were incubated with different dose EEDQ2 preparations for 24h to measure the IC_50_.

### Mitochondrial transmembrane potential (ΔΨm) assay

JC–1 dye is a fluorescent probe that is widely used in the detection of mitochondrial membrane potential. JC-1 dye shows potential dependent accumulation within the mitochondria[21].

The cells were planted in 6-wells plate 2*10^5^cells per well, cultured for 24h, and then treated with 15μg/ml of EEDQ2, LP-EEDQ2, or Tf-LP-EEDQ2. After 12h, the cells were washed with PBS, incubated with 10 μg/ml of JC-1 dye in PBS (pH7.4) at 37°C in the dark for 10min. Inverted fluorescent microscope was used to visualize the mitochondrial depolarization level, the mitochondrial depolarization level was quantitatively analyzed by imaging software Image J.

### Assessment the generation level of reactive oxygen species (ROS)

Cells were seeded in 6 well plates at a density of 2 x 10^5^ cells per well and 15μg/ml agents (EEDQ2, LP-EEDQ2 or Tf-LP-EEDQ2) were added and the cells incubated for 12h. Then, the cells were incubated with 10μM 2'-7'-dichlorodihydrofluorescein diacetate (DCFH-DA, Sigma-Aldrich, USA) for 10 min in darkness at 37°C[22, 23]. Then, images were obtainedby Inverted fluorescent microscope.

### Confocal microscopy and cellular internalization analysis

To determine whether EEDQ2 can be effectively internalized into cells[24], we used Tf-LPs as a delivery vehicle to deliver the drug. Cells were seeded in a 35mm diameter glass bottom culture dishes at a density of 1*10^5^ cells per well and cultured for another 24h. Then culture medium was replaced by LP-EEDQ2 or Tf-LP-EEDQ2 dissolved in basic DMEM medium and cultured for another 4h. All the LPs were labeled with sulforhodamine B and NBD-DOPE. And then the cells were washed with PBS and fixed by 350μL of 4% paraformaldehyde for 10 min, cells were washed again. Two μg/ml DAPI dye was used to stain the nucleus for 10min. Then, dye solution was replaced by PBS, the cells were immediately characterized using a Zeiss 710 LSMNLO Confocal Microscope (Carl Zeiss; Jena, Germany).

### Uptake of LP-EEDQ2 and Tf-LP-EEDQ2

Cells were seeded in a 6-well plate to culture 24h at 2*10^5^cells per well. Then culture medium was replaced by LP-EEDQ2, Tf-LP-EEDQ2 or Tf-LP-EEDQ2 in DMEM medium and the cells were cultured for another 4h. All the LPs were labeled with sulforhodamine B and NBD-DOPE. The cells were then washed with PBS and harvested with 150μL trypsin and fixed in 350μL 4% formaldehyde solution. The cells uptake of liposomes were measured by a EPICS XL flow cytometer[25] (Beckman Coulter Corp., Brea, CA, USA).

### Statistical analysis

The statistical significance of data was analyzed by Student’s t-test. P values <0.05 and <0.01 mean significant difference and extremely significant difference. The results were shown as mean ±SD.

## Results and discussion

### In vitro anticancer activity

**Fig 2:**
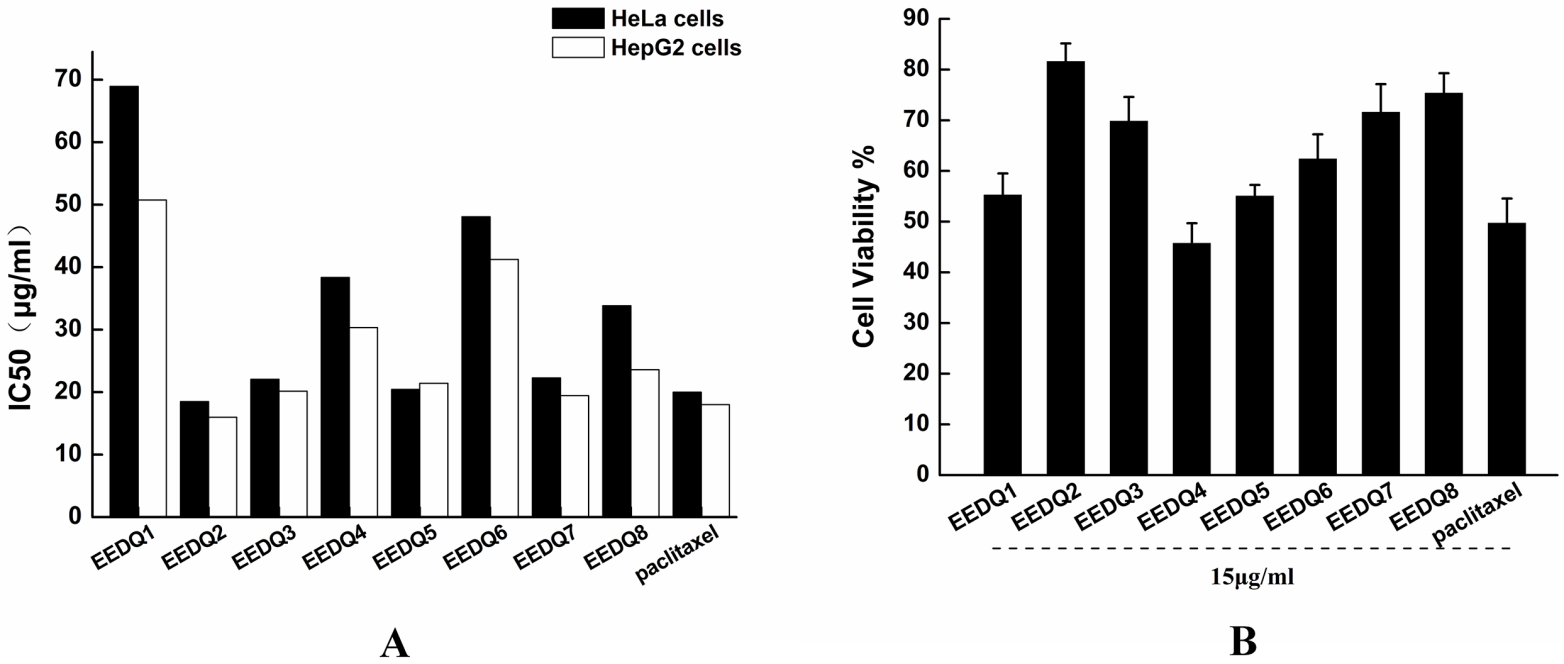
Cytotoxicity of EEDQ derivatives. (A) IC_50_ of the novel EEDQ derivatives in HeLa and Hep G2 cell lines. (B) Cytotoxicity of 15μg/ml EEDQ derivatives against normal cells 6HEK-293T. Data represent mean ± SD (n = 6).

We synthesized a serious of EEDQ derivatives. The cytotoxicity of the novel EEDQ derivatives was tested in HeLa, Hep G2 and 6HEK-293T cell lines using MTT assay. IC_50_ valued in HeLa and Hep G2 cell lines were shown in Fig 2. All EEDQ compounds showed certain antitumor activity. In two cancer lines, EEDQ2, 3, 5, 7, 8 showed similar level of cytotoxicity to paclitaxel.

EEDQ derivatives at 15ug/ml had low cell cytotoxicity effect in normal cells compared to paclitaxel. This suggests that EEDQ derivatives can induce tumor cell death and have low toxicity to normal cells. We chose EEDQ2 for further evaluation based on its high cytotoxicity to cancer cells and relative low cytotoxicity to HEK-293T cells.

### Characterization of Tf-LP-EEDQ2

Although EEDQ2 had promising tumor cell cytotoxicity, it suffers from low water solubility, high clearance rate in plasma and low tumor accumulation. Therefore, Tf-LPs were used as a carrier to overcome these problems. The fate of Tf-LP-EEDQ2 was determined by the surface properties of LPs. The PEG coating on the LPs should produce long circulation in plasma. Although TfR is widely expressed in normal cells, the expression level of TfR in malignant tumor cells is typically 100-fold higher than normal cells. Tumor cells can actively take up Tf-LPs by TfR mediated pathway.

Zeta potential and particle size of EEDQ2, blank Tf-LP, LP-EEDQ2 and Tf-LP-EEDQ2 are summarized in Table 1. The particle size was about 100nm, which allows LPs passively target tumors through the enhanced permeability and retention (EPR) effect. A slight negative charge onto LPs surface guarantees that LPs are not nonspecific adsorbed by normal tissue cells in the process of circulation.

**Table 1:** Characterization of zeta potential and particle size.

	EEDQ2	Blank Tf-LP	LP-EEDQ2	Tf-LP-EEDQ2
**Particle size**	N/A	95.26±7.2nm	92.2±2.1nm	103.4±2.8nm
**ζ-Potential**	5.73±0.43mv	-7.55±0.98mv	-3.12±0.43mv	-3.36±0.71mv

N/A, not applicable

**Fig 3:**
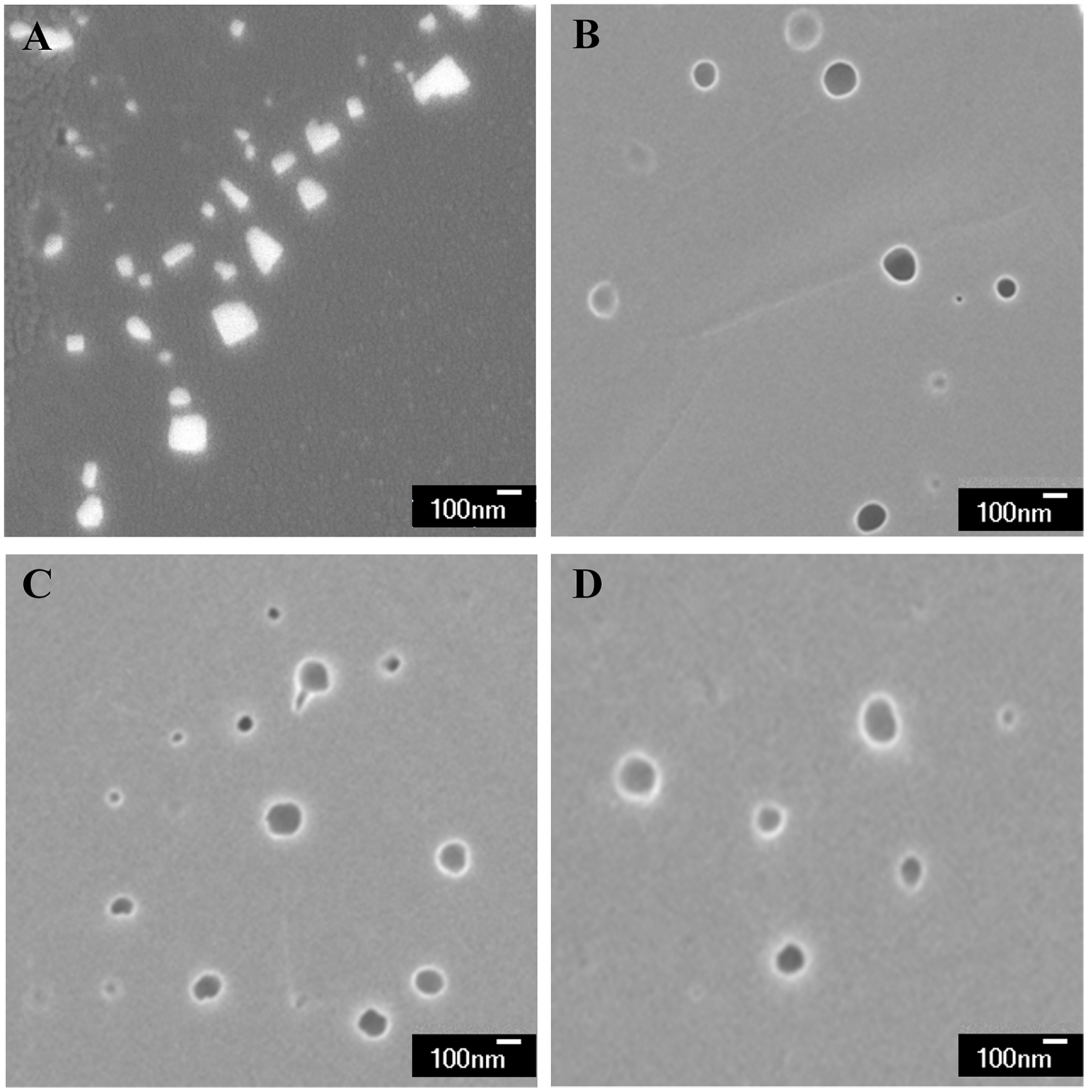
FE-SEM images of EEDQ2 preparations. (A) FE-SEM image of EEDQ2 (×30,000), (B) FE-SEM image of blank Tf-LP (× 30,000), (C) FE-SEM image of LP-EEDQ2 (× 30,000), (D) FE-SEM image of Tf-LP-EEDQ2 (× 30,000).

FE-SEM result was shown in Fig 3. LPs appeared spherical with uniform size and smooth surface. In contrast, the FE-SEM image of EEDQ2 showed irregular shape with higher brightness. The structure resembled a flaky crystal, which was significantly different from Tf-LP-EEDQ2.

We firstly established a HPLC method to test the concentration of EEDQ2. EEDQ2 has a significant absorption peak at the wavelength 240nm. We used methanol and water (60:40) to separate EEDQ2. In the range of 0.1–20μg/ml, EEDQ2 showed good linear correlation with least-squares linear regression constants (R2) that is higher than 0.99. The EE% of EEDQ2 was about 99%.

### Drug release profiles

S1 Fig shows the *in vitro* release profiles of free EEDQ2, LP-EEDQ2, Tf-LP-EEDQ2 in PBS containing 0.5% Tween 80 at 37°C. Free EEDQ2 showed rapid release relative to LP preparations. Free EEDQ2 was 90% released within 4h while LP-EEDQ2 and Tf-LP-EEDQ2 were only released at about 50%. LP-EEDQ2 and Tf-LP-EEDQ2 showed release of 65%-70% after 24h. The release profiles of LP-EEDQ2 and Tf-LP-EEDQ2 were similar. This meant that Tf incorporation into LPs did not affect release rate. The above data proved that LPs of EEDQ2 could facilitate sustained release.

### Cytotoxicity assay

**Fig 4:**
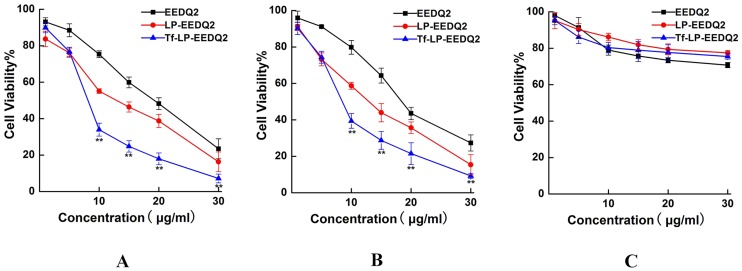
MTT viability assay of EEDQ LPs. Viability of Hela cells (A), HepG cells (B) and HEK-293T cells (C)after treatment with EEDQ2, LP-EEDQ2 or Tf-LP-EEDQ2 at the concentration of 0–30 mg/ ml for 24 h. The cells treated with PBS were used as control. (*p<0.05, **p<0.01: LP-EEDQ2 VS Tf-LP-EEDQ2).

We next explored the cytotoxicity of EEDQ2, LP-EEDQ2 or Tf-LP-EEDQ2 at different concentrations and time of exposure. In this study, tumor cell lines HeLa, HepG2 and normal cell lines HEK-293T were used to value the cytotoxic effects of EEDQ2, LP-EEDQ2 and Tf-LP-EEDQ2. The cytotoxicity results of agents in different concentration were shown in Fig 4. The IC_50_ values were similar between EEDQ2 and LP-EEDQ2 in tumor cell lines HeLa (18.55μg/ml, 10.01μg/ml) and HepG2 (14.53μg/ml, 10.44μg/ml). The IC_50_ value of Tf-LP-EEDQ2 in HeLa (6.49μg/ml), HepG2 (7.12μg/ml) were significant lower than those of EEDQ2 and LP-EEDQ2. This means that Tf-LP-EEDQ2 had greater anticancer activity. But in normal cell lines HEK-293T, the cytotoxicity of LP-EEDQ2 and Tf-LP-EEDQ2 at 15ug/ml were similar to those of EEDQ2. We predicted that Tf-LPs can enhance tumor cells uptake EEDQ2 through Tf-mediated pathway.

**Fig 5:**
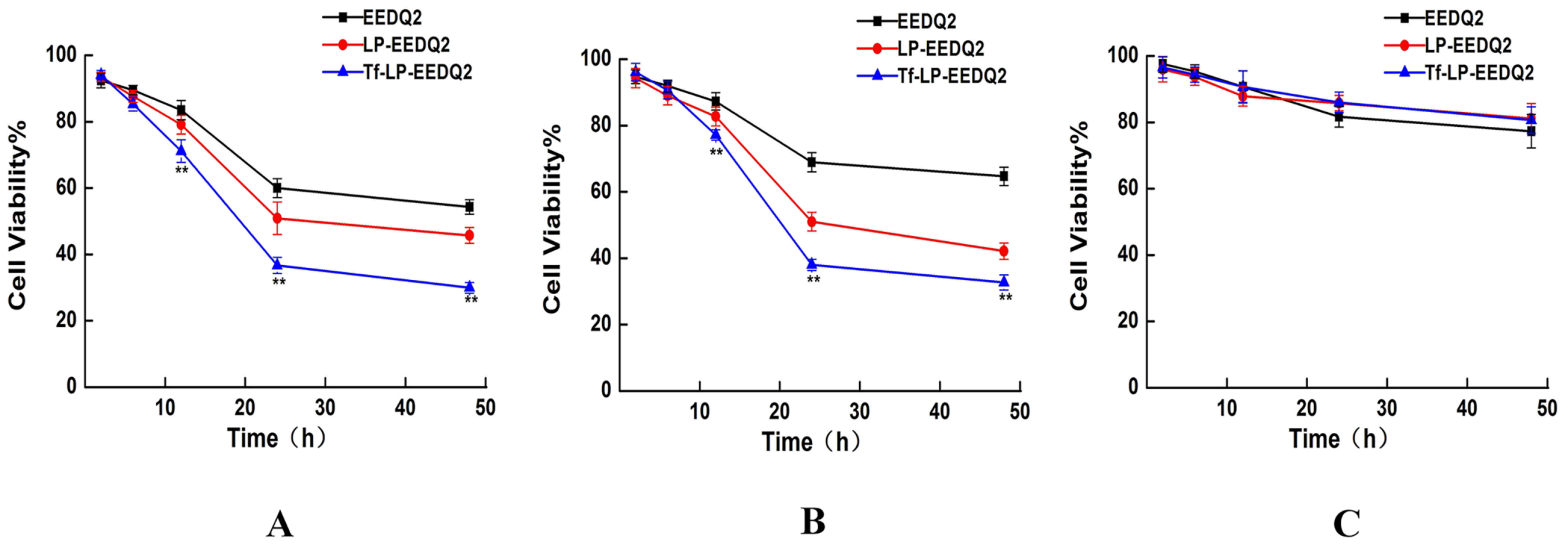
MTT viability assay on EEDQ LPs after different exposure time. Viability of Hela cells (A), Hep G2 cells (B) and HEK-293T cells (C) after treatment with EEDQ2, LP-EEDQ2 or Tf-LP-EEDQ2 for different length of time. Cells treated with PBS were used as control. (*p<0.05, **p<0.01: LP-EEDQ2 VS Tf-LP-EEDQ2).

We studied the time depended cytotoxicity of 15μg/ml EEDQ2, LP-EEDQ2 and Tf-LP-EEDQ2. When the cancer cells were exposed to EEDQ2 preparations for 24 h, EEDQ2 preparations reached the maximum effect (Fig 5).

### Mitochondrial membrane potential changes

Cellular apoptosis is often coupled with the destruction of mitochondrial membrane potential. We expect that EEDQ2 could induce cancer cells apoptosis. Therefore, we explored mitochondrial membrane potential transformation by JC-1 dyeing[26].

JC-1 dye accumulates in mitochondria, which depends on mitochondrial membrane potential. In physiologically polarized cells, JC-1 accumulated in the mitochondrial to form a polymer, which gives off a strong red fluorescence. Upon unhealthy mitochondria membrane potential drop or loss, JC-1 only exists in cytoplasm to produce green fluorescence with in form of monomer. So the color change can directly reflect the change of mitochondrial membrane potential. Mitochondrial depolarization can be measured through the proportion of red/green fluorescence intensity.

**Fig 6:**
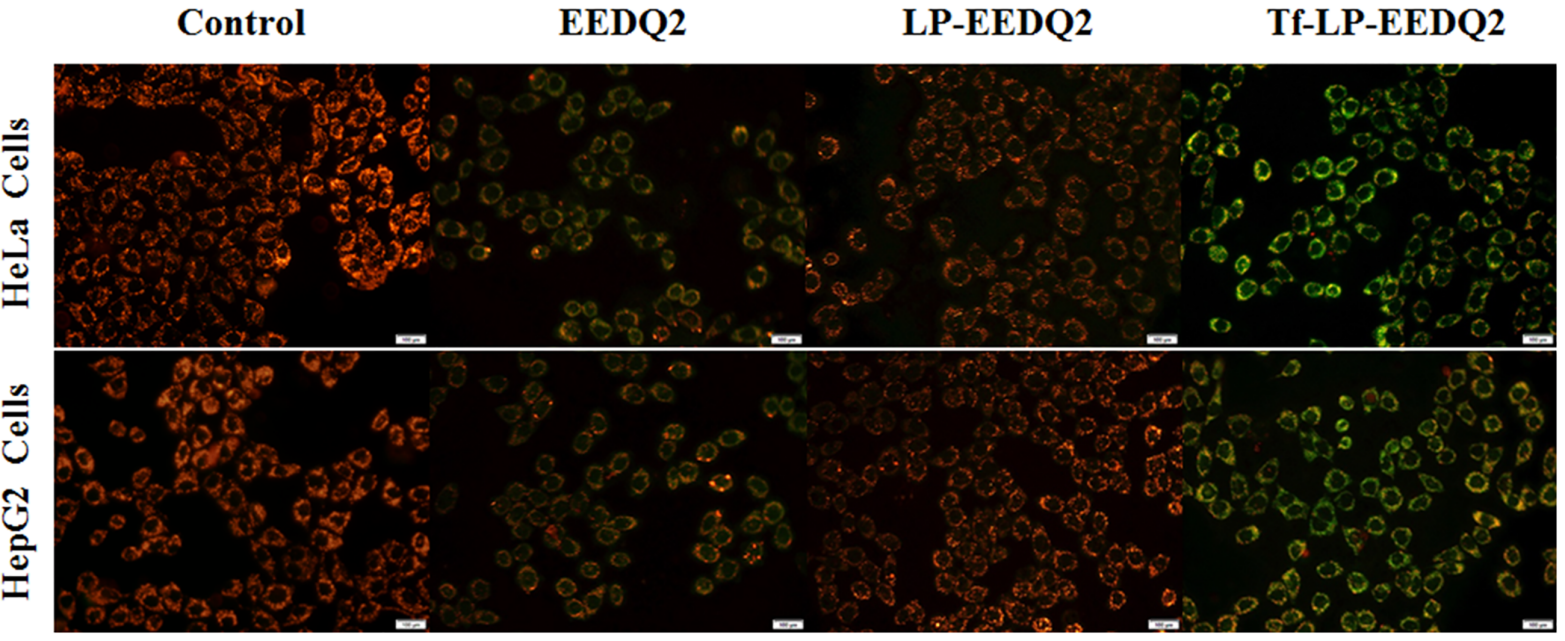
Effect of EEDQ2 on cellular mitochondria. The tumor cells (HeLa and HepG2 cells) were treated with EEDQ2, LP-EEDQ2 or Tf-LP-EEDQ2, the level of mitochondrial depolarization showed by fluorescence.

The results of mitochondrial membrane potential changes in two tumor cell lines (HeLa and HepG2 cells) treated with EEDQ2, LP-EEDQ2 or Tf-LP-EEDQ2 were shown in Fig 6 and S2 Fig. The red/green fluorescence intensity was qualitative analyzed in the tumor cells (HeLa and HepG2 cells) after treatment with EEDQ2, LP-EEDQ2 or Tf-LP-EEDQ2. The data showed that EEDQ2 induced mitochondrial membrane potential depolarization (Fig 6), as shown by increased green fluorescence. The red/green fluorescence merge image of two tumor cell lines treated with Tf-LP-EEDQ2 induced stronger green fluorescence than EEDQ2 and LP-EEDQ2, suggesting that Tf-modified LPs enhanced the cytotoxic effect of EEDQ2. Further quantitative analysis the red/green fluorescence intensity ratio (S2 Fig) showed that the fluorescence ratio of Tf-LP-EEDQ2 was lowest (HeLa = 0.59, HepG2 = 0.6). Tf-LP-EEDQ2 can significantly enhance the degree of mitochondrial membrane potential depolarization than other EEDQ2 formulations groups. To sum up, EEDQ2 might induce tumor cells apoptosis by changing the mitochondrial membrane potential.

### Assessment the generation level of reactive oxygen species (ROS)

Mitochondria act an important role in the process of cell proliferation and metabolism. Besides producing ATP, they were also involved in programmed cell death through releasing ROS as a secondary messenger in cellular signaling. High level ROS could result mitochondrial dysfunction[27]. This is a virtuous cycle to make cancer cells apoptosis.

**Fig 7:**
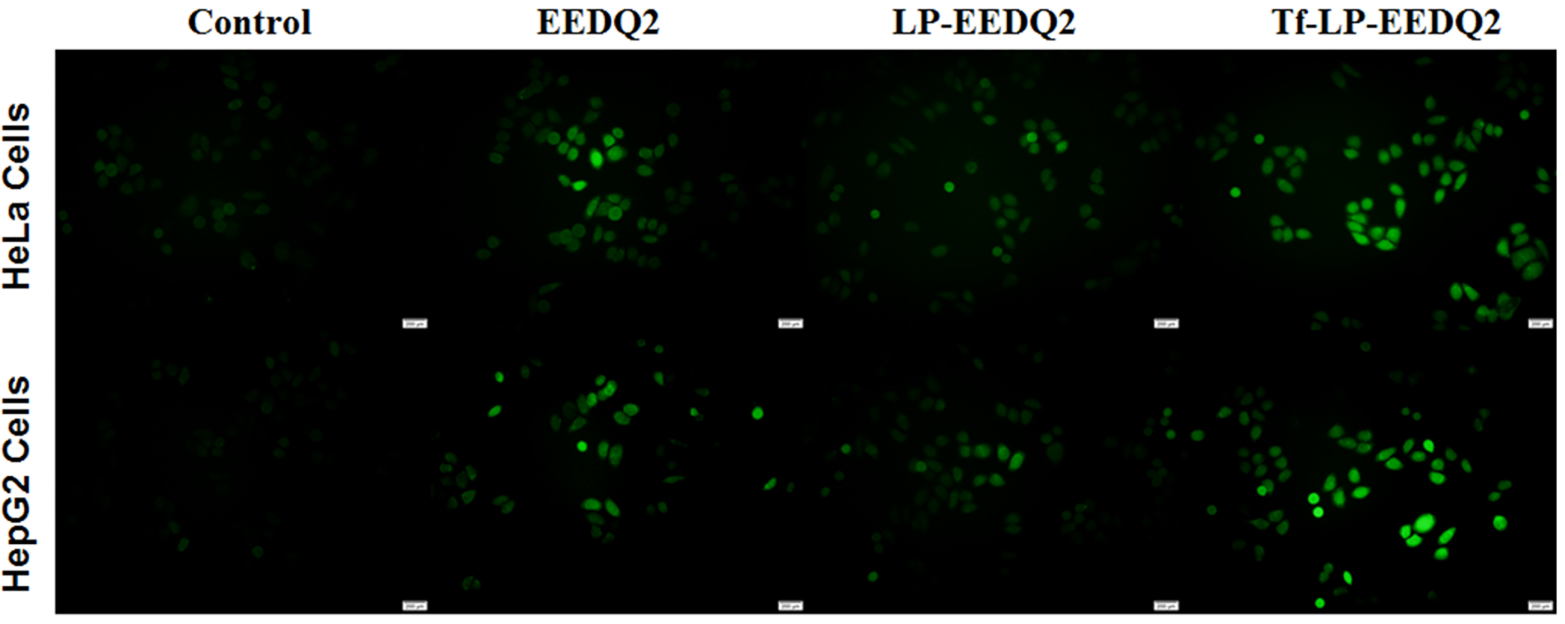
The ROS level of cancer cells after incubation with EEDQ2, LP-EEDQ2 or Tf-LP-EEDQ2.

The ROS level of two cancer cell lines was shown in Fig 7 after treatment with EEDQ2, LP-EEDQ2 or Tf-LP-EEDQ2. We observed that EEDQ2 preparations induced more ROS production in cancer cells from fluorescence image. Tf-LP-EEDQ2 showed the brightest green fluorescence. The fluorescence intensity was quantitatively analyzed in S3 Fig, which suggests that it had the greatest cytotoxicity. We believe that the enhanced effect of Tf-LP-EEDQ2 was due to targeting of Tf-modified LPs.

### Confocal microscopy evaluation of Tf-LP-EEDQ2 cellular uptake

**Fig 8:**
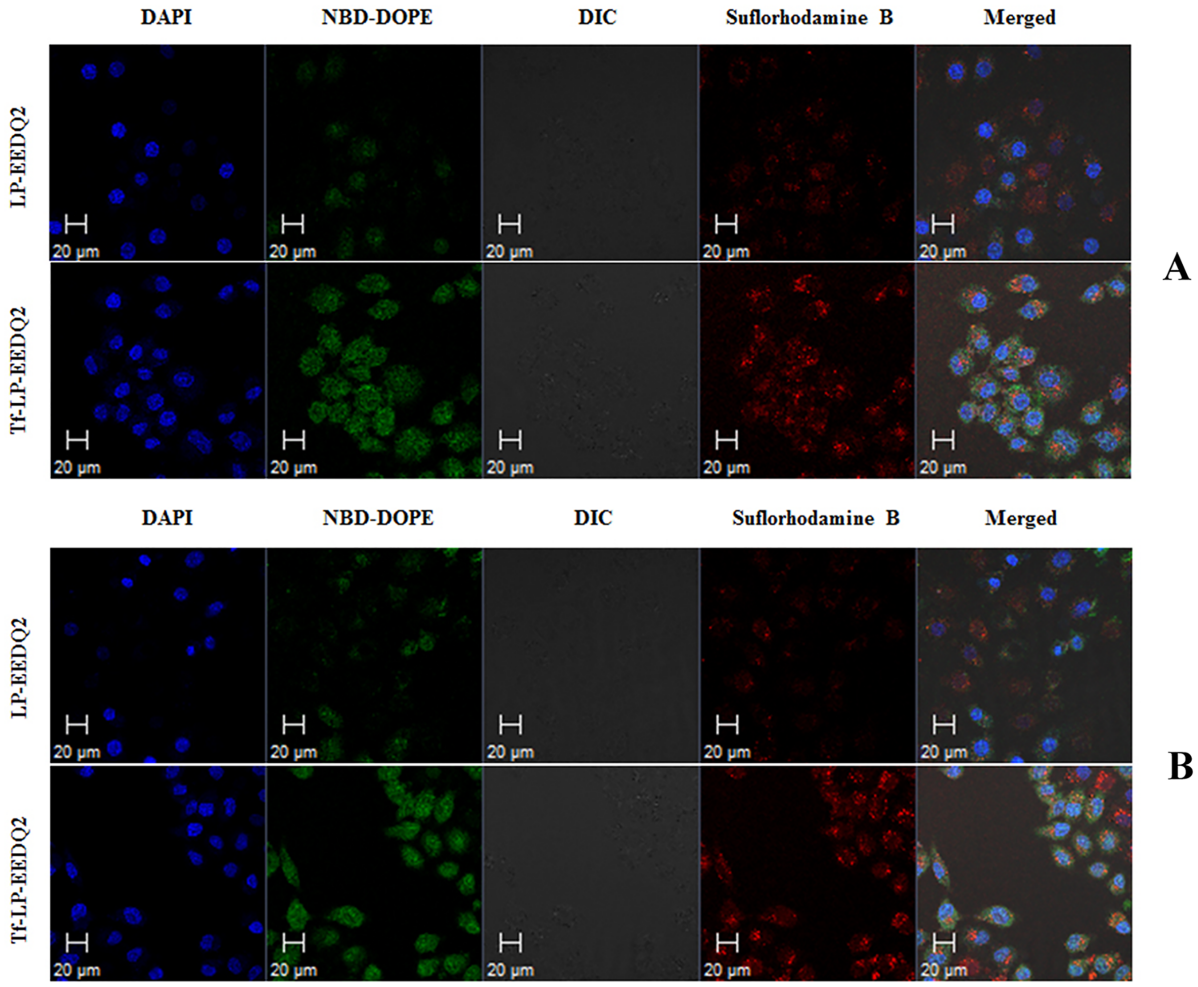
Intracellular localization of LP-EEDQ2 and Tf-LP-EEDQ2. Confocal microscopy analysis was performed after 4 h of incubation of LP-EEDQ2 and Tf-LP-EEDQ2 with HeLa cells (A) and HepG2 cells (B).

Tumor uptake of LP-EEDQ2 and Tf-LP-EEDQ2 was investigated by confocal microscopy (Fig 8). The two tumor cells were treated with NBD-DOPE and sulforhodamine B labeled LPs. The pictures suggested that LPs were localized within the cytoplasm and nuclei of cells and high fluorescence intensity of Tf-LP-EEDQ2 indicates better internalization effect. Non-targeted LPs delivered a relatively small number of LPs to cells. The above results mean that (Tf)-modified liposomes is an efficient delivery carrier for EEDQ2.

### Effect of Tf-LP-EEDQ2 on cellular uptake

**Fig 9:**
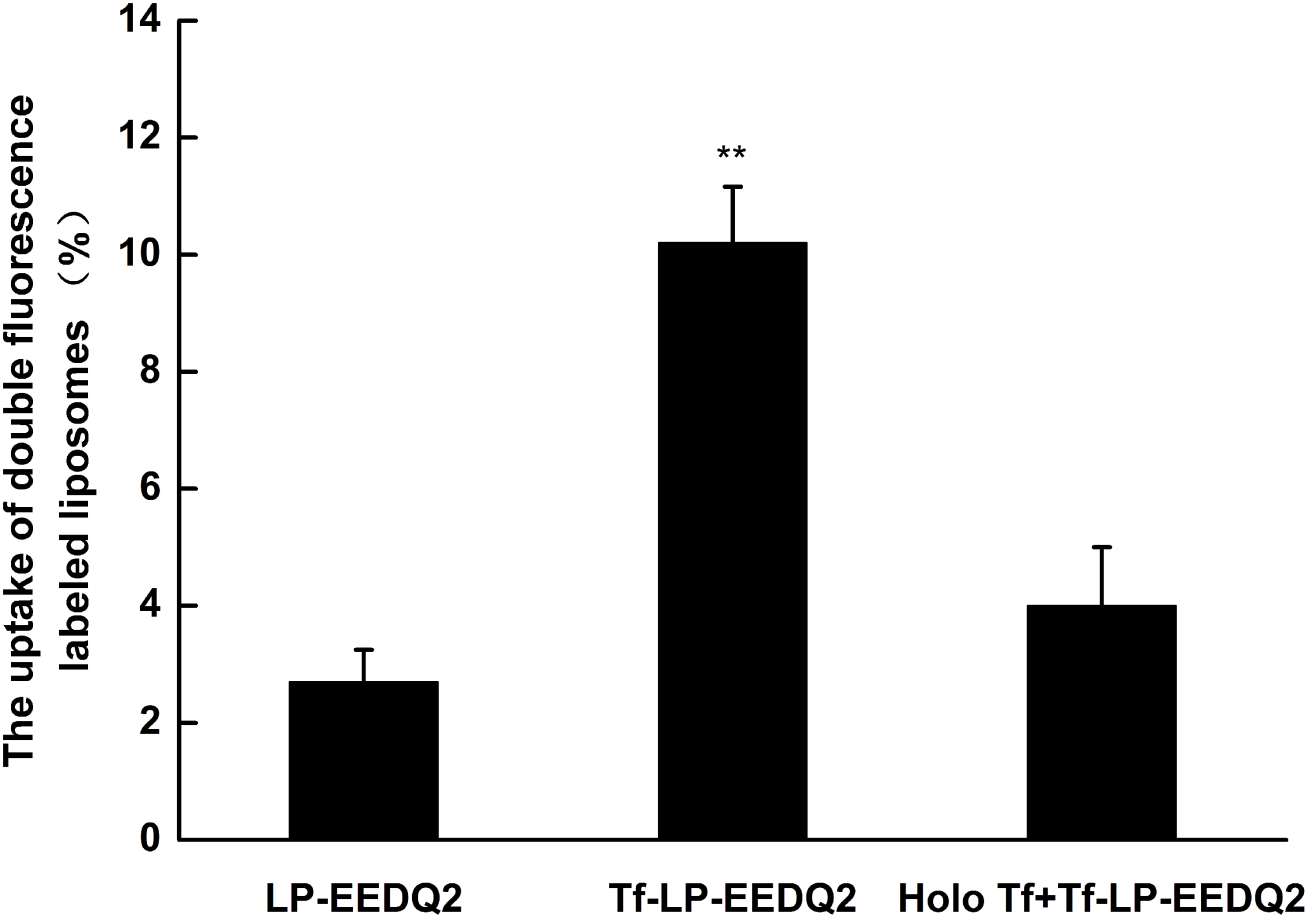
Cellular uptake of LPs. HeLa cells were treated with LP-EEDQ2, Tf-LP-EEDQ2, or Tf-LP-EEDQ2 with holo-Tf *in vitro* and then measured by flow cytometery. Data represent the mean± standard deviation (n = 3). Significantly different at **p<0.01 (Tf-LP-EEDQ2 versus Tf-LP-EEDQ2 + holo-Tf).

Tumor cells uptake was evaluated by incubating Hela cells after treatment with various LP-EEDQ2 or Tf-LP-EEDQ2 or Tf-LP-EEDQ2 with holo-Tf (holo-Tf was added 2h earlier than Tf-LP-EEDQ2). Flow cytometry was used to perform quantitative analysis of the cellular uptake of various liposomal preparations containing EEDQ2. Results are shown in Fig 9. The aqueous phase of LPs was labeled by rhodamine B and lipid phase was labeled using NBD. The uptake of NBD-DOPE and sulforhodamine B LP-EEDQ2 was 2.7%, but when we used Tf to modify LPs, the uptake rapid increased to 10.2%. In order to determine whether the uptake of Tf-LP-EEDQ2 was mediated by Tf, we added excess free Tf to block TfR before adding Tf-LP-EEDQ2. The uptake of Tf-LP-EEDQ2 with free holo-Tf significantly decreased from 10.2% to 4%. These data indicate that Tf-LP-EEDQ2 mediated more efficient uptake of EEDQ2 *in vitro*.

## Conclusions

On the basis of our previous studies, we used EEDQ as a basic pharmacophore to synthesize a series of new derivatives for cancer treatment. Studies have indicated that EEDQ derivatives had antitumor activity through hanging the mitochondrial membrane potential and inducing ROS. In order to improve cells uptake, we selected EEDQ2 with high tumor cytotoxicity and low normal cells toxicity to build Tf-LPs. The results suggest EEDQ2 as a novel agent that can be developed into potential anticancer chemotherapeutic in the future.

In future work, we plan to further evaluate the pharmacodynamics and pharmacokinetics of Tf-LP-EEDQ2 *in vivo*.

## Supporting information

S1 Fig Drug release profiles of LP-EEDQ2, Tf-LP-EEDQ2 and free EEDQ2. *In vitro* release of LP-EEDQ2, Tf-LP-EEDQ2 and free EEDQ2 within 24h in PBS containing 0.5% Tween 80, data was measured 3 times and showed as mean±SD.
(TIF)

S2 Fig Mitochondrial membrane potential changing of cancer cells after treatment with EEDQ2, LP-EEDQ2 or Tf-LP-EEDQ2. The red/green fluorescence intensity ratio in the tumor cells (HeLa and HepG2 cells) after treatment with EEDQ2, LP-EEDQ2 or Tf-LP-EEDQ2 were used to show the level of mitochondrial depolarization. Significantly different at **p<0.01 (Tf-LP-EEDQ2 versus LP-EEDQ2).
(TIF)

S3 Fig The ROS generation of cancer cells after treatment with EEDQ2, LP-EEDQ2 or Tf-LP-EEDQ2. The fluorescence intensity in the tumor cells(HeLa and HepG2 cells) after treatment with EEDQ2, LP-EEDQ2 or Tf-LP-EEDQ2 used to quantitatively analyze the ROS generation. Significantly different at **p<0.01 (Tf-LP-EEDQ2 versus LP-EEDQ2).
(TIF)
